# The genome sequence of the hairy rockcress, *Arabis hirsuta* (L.) Scop. (Brassicales: Brassicaceae)

**DOI:** 10.12688/wellcomeopenres.24571.1

**Published:** 2025-07-25

**Authors:** Markus Ruhsam, Peter M Hollingsworth, Royal Botanic Garden Edinburgh Genome Acquisition Lab, Darwin Tree of Life Barcoding collective, Plant Genome Sizing collective, Wellcome Sanger Institute Tree of Life Management, Samples and Laboratory team, Wellcome Sanger Institute Scientific Operations: Sequencing Operations, Wellcome Sanger Institute Tree of Life Core Informatics team, Tree of Life Core Informatics collective, Darwin Tree of Life Consortium

**Affiliations:** 1 Royal Botanic Garden Edinburgh, Edinburgh, Scotland, UK

**Keywords:** Arabis hirsuta, Hairy rockcress, genome sequence, chromosomal, Brassicales

## Abstract

We present a genome assembly from a specimen of *Arabis hirsuta* (Hairy rockcress; Streptophyta; Magnoliopsida; Brassicales; Brassicaceae). The assembly contains two haplotypes with total lengths of 582.67 megabases and 576.85 megabases. Most of haplotype 1 (99.96%) is scaffolded into 16 chromosomal pseudomolecules. Haplotype 2 was assembled to scaffold level. The mitochondrial and plastid genome assemblies have lengths of 342.94 kilobases and 153.77 kilobases, respectively.

## Species taxonomy

Eukaryota; Viridiplantae; Streptophyta; Streptophytina; Embryophyta; Tracheophyta; Euphyllophyta; Spermatophyta; Magnoliopsida; Mesangiospermae; eudicotyledons; Gunneridae; Pentapetalae; rosids; malvids; Brassicales; Brassicaceae; Arabideae; *Arabis*; *Arabis hirsuta* (L.) Scop. (NCBI:txid78191)

## Background

*Arabis hirsuta* or the hairy rockcress is a 30–60 cm tall, tufted biennial (sometimes perennial), growing on lime rich soils in bare places, on walls and in grassland. It is native to Europe and parts of Asia and locally common in the UK ( Stace *et al.*, 2019). *A. hirsuta* is an aggregate species which contains several closely related taxa associated with a highly debated taxonomy among authors during the last hundred years (see Karl & Koch, 2014 and references therein). There is a high variability in morphological characters such as plant size, leaf number and shape, and indumentum, which makes species identification in the *A. hirsuta* aggregate challenging ( Titz, 1969). The aggregate currently consists of six species ( Karl & Koch, 2014): *A. hirsuta*, *A. sagittata* (Bertol.) DC., *A. planisiliqua* (Pers.) Rchb., *A. nemorensis* (J.P.Wolff ex Hoffm.) W.D.J. Koch, *A. allionii* DC., and *A. sudetica* Tausch. In the UK, only *A. hirsuta* is recorded ( Stace *et al.*, 2019).

Hairy rockcress is reported to be autogamous but pseudogamous apomixis could not be excluded, so more work is needed to clarify the breeding system of this species ( Roy, 1995). *Arabis hirsuta* is a tetraploid species (2 *n* = 32) with a base chromosome number of *x* = 8 ( Karl & Koch, 2014; Koch *et al.*, 1999; Titz, 1972). This chromosome count of 2 *n* = 32 has been reported from five different localities in Britain and Ireland ( Gornall & Bailey, 1993). It may have an allotetraploid origin with the possible parents being *A. sagittata* (Bertol.) DC. and *A. ciliata* Clairv. ( Titz, 1972).

**Figure 1.**
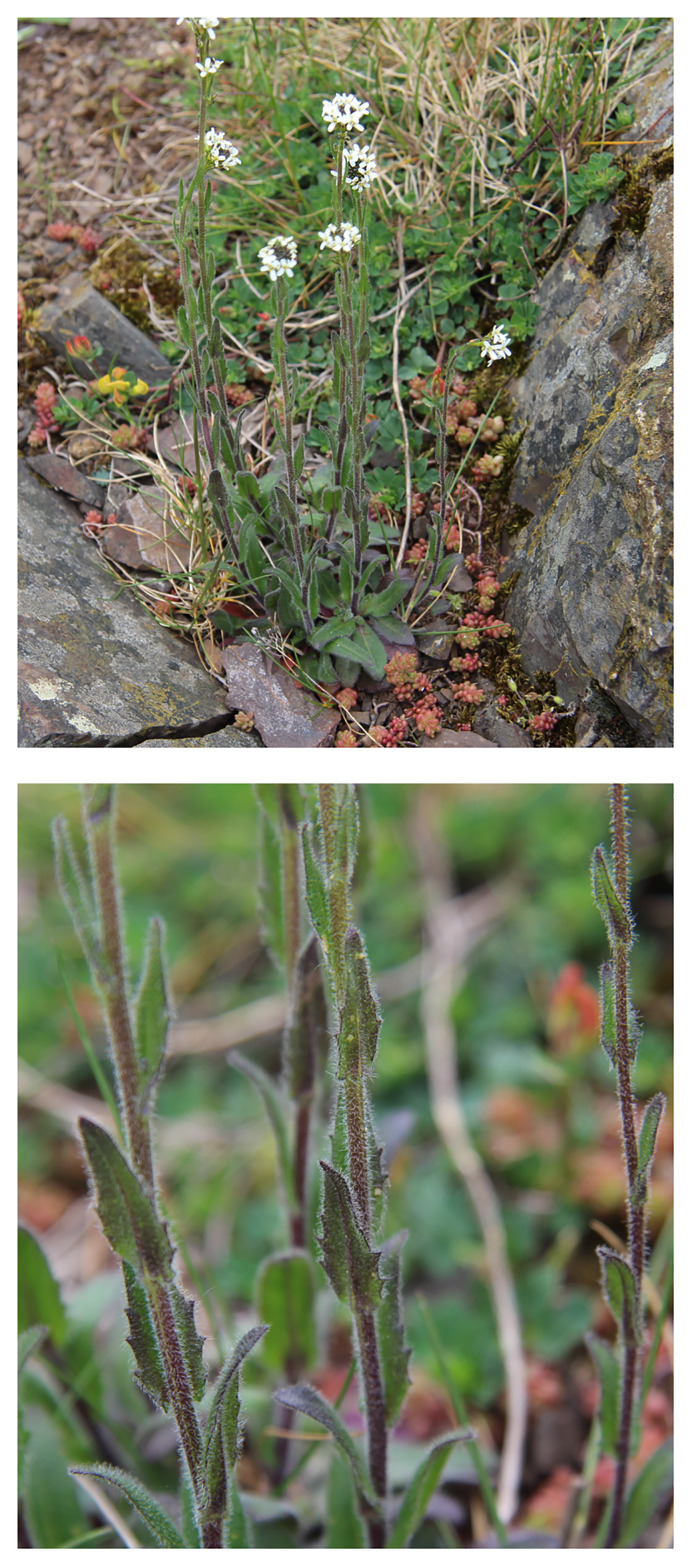
Photographs of the *Arabis hirsuta* (ddAraHirs1) specimen from which samples were taken for genome sequencing. Top: whole plant, Bottom: close-up of the leaves.

We present a chromosomally complete genome sequence for *Arabis hirsuta*, which may help to resolve the complex evolutionary history of this species complex. This genome was assembled using the Tree of Life pipeline from a specimen collected from Hermitage, Edinburgh, United Kingdom ( Figure 1).

## Methods

### Sample acquisition, flow cytometry and DNA barcoding

The *Arabis hirsuta* specimen used for genome sequencing (specimen ID EDTOL05514, ToLID ddAraHirs1; Figure 1) was collected from Hermitage, Edinburgh, United Kingdom (latitude 55.9196, longitude –3.1877) on 2023-05-17. The specimen was collected and identified by Markus Ruhsam (Royal Botanic Garden Edinburgh). The herbarium voucher associated with the sequenced plant is DMR301 and is deposited in the herbarium of RBG Edinburgh (E) https://data.rbge.org.uk/herb/E01358078. Sample metadata was collected in line with the recommended standards for the Darwin Tree of Life project ( Lawniczak *et al.*, 2022).

The genome size was estimated by flow cytometry using propidium iodide as the fluorochrome and following the ‘one-step’ method as outlined in Pellicer *et al*. (2021). The General Purpose Buffer (GPB) supplemented with 3% PVP and 0.08% (v/v) beta-mercaptoethanol was used for isolation of nuclei ( Loureiro *et al.*, 2007).

The genome size was estimated by flow cytometry using the fluorochrome propidium iodide and following the ‘one-step’ method as outlined in Pellicer *et al*. (2021). For this species, the General Purpose Buffer (GPB) supplemented with 3% PVP and 0.08% (v/v) beta-mercaptoethanol was used for isolation of nuclei ( Loureiro *et al.*, 2007), and the internal calibration standard was *Solanum lycopersicum* ‘Stupiké polní rané’ with an assumed 1C-value of 968 Mb ( Doležel *et al.*, 2007).

The initial identification was verified by an additional DNA barcoding process according to the framework developed by Twyford *et al*. (2024). Part of the plant specimen was preserved in silica gel desiccant ( Chase *et al.*, 1991). DNA extracted from the dried plant was amplified by PCR for standard barcode markers, with the amplicons sequenced and compared to public sequence databases including GenBank and the Barcode of Life Database (BOLD) ( Ratnasingham & Hebert, 2007). Following whole genome sequence generation, the relevant DNA barcode region was also used alongside the initial barcoding data for sample tracking at the WSI ( Twyford *et al.*, 2024). The standard operating procedures for Darwin Tree of Life barcoding are available on protocols.io.

### Nucleic acid extraction

Protocols for high molecular weight (HMW) DNA extraction developed at the Wellcome Sanger Institute (WSI) Tree of Life Core Laboratory are available on protocols.io ( Howard *et al.*, 2025). The ddAraHirs1 sample was weighed and triaged to determine the appropriate extraction protocol. The leaf tissue was homogenised by cryogenic bead beating. HMW DNA was extracted using the Automated Plant MagAttract v2 protocol. DNA was sheared into an average fragment size of 12–20 kb following the Megaruptor®3 for LI PacBio protocol. Sheared DNA was purified by manual SPRI (solid-phase reversible immobilisation). The concentration of the sheared and purified DNA was assessed using a Nanodrop spectrophotometer and Qubit Fluorometer and Qubit dsDNA High Sensitivity Assay kit. Fragment size distribution was evaluated by running the sample on the FemtoPulse system. For this sample, the final post-shearing DNA had a Qubit concentration of 7.48 ng/μL and a yield of 351.56 ng, with a fragment size of 12.8 kb. The 260/280 spectrophotometric ratio was 2.09, and the 260/230 ratio was 3.95.

### PacBio HiFi library preparation and sequencing

Library preparation and sequencing were performed at the WSI Scientific Operations core. Libraries were prepared using the SMRTbell Prep Kit 3.0 (Pacific Biosciences, California, USA),according to the manufacturer’s instructions. The kit includes reagents for end repair/A-tailing, adapter ligation, post-ligation SMRTbell bead clean-up, and nuclease treatment. Size selection and clean-up were performed using diluted AMPure PB beads (Pacific Biosciences). DNA concentration was quantified using a Qubit Fluorometer v4.0 (ThermoFisher Scientific) and the Qubit 1X dsDNA HS assay kit. Final library fragment size was assessed with the Agilent Femto Pulse Automated Pulsed Field CE Instrument (Agilent Technologies) using the gDNA 55 kb BAC analysis kit.

The sample was sequenced using the Sequel IIe system (Pacific Biosciences, California, USA). The concentration of the library loaded onto the Sequel IIe was in the range 40–135 pM. The SMRT link software, a PacBio web-based end-to-end workflow manager, was used to set-up and monitor the run, and to perform primary and secondary analysis of the data upon completion.

### Hi-C

***Sample preparation and crosslinking***

The Hi-C sample was prepared from 20–50 mg of frozen leaf tissue of the ddAraHirs1 sample using the Arima-HiC v2 kit (Arima Genomics). Following the manufacturer’s instructions, tissue was fixed and DNA crosslinked using TC buffer to a final formaldehyde concentration of 2%. The tissue was homogenised using the Diagnocine Power Masher-II. Crosslinked DNA was digested with a restriction enzyme master mix, biotinylated, and ligated. Clean-up was performed with SPRISelect beads before library preparation. DNA concentration was measured with the Qubit Fluorometer (Thermo Fisher Scientific) and Qubit HS Assay Kit. The biotinylation percentage was estimated using the Arima-HiC v2 QC beads.

***Hi-C library preparation and sequencing***

Biotinylated DNA constructs were fragmented using a Covaris E220 sonicator and size selected to 400–600 bp using SPRISelect beads. DNA was enriched with Arima-HiC v2 kit Enrichment beads. End repair, A-tailing, and adapter ligation were carried out with the NEBNext Ultra II DNA Library Prep Kit (New England Biolabs), following a modified protocol where library preparation occurs while DNA remains bound to the Enrichment beads. Library amplification was performed using KAPA HiFi HotStart mix and a custom Unique Dual Index (UDI) barcode set (Integrated DNA Technologies). Depending on sample concentration and biotinylation percentage determined at the crosslinking stage, libraries were amplified with 10–16 PCR cycles. Post-PCR clean-up was performed with SPRISelect beads. Libraries were quantified using the AccuClear Ultra High Sensitivity dsDNA Standards Assay Kit (Biotium) and a FLUOstar Omega plate reader (BMG Labtech).

Prior to sequencing, libraries were normalised to 10 ng/μL. Normalised libraries were quantified again and equimolar and/or weighted 2.8 nM pools. Pool concentrations were checked using the Agilent 4200 TapeStation (Agilent) with High Sensitivity D500 reagents before sequencing. Sequencing was performed using paired-end 150 bp reads on the Illumina NovaSeq X.

### Genome assembly

Prior to assembly of the PacBio HiFi reads, a database of *k*-mer counts ( *k* = 31) was generated from the filtered reads using FastK. GenomeScope2 ( Ranallo-Benavidez *et al.*, 2020) was used to analyse the *k*-mer frequency distributions, providing estimates of genome size, heterozygosity, and repeat content.

The HiFi reads were assembled using Hifiasm in Hi-C phasing mode ( Cheng *et al.*, 2021; Cheng *et al.*, 2022), producing two haplotypes. Hi-C reads ( Rao *et al.*, 2014) were mapped to the primary contigs using bwa-mem2 ( Vasimuddin *et al.*, 2019). Contigs were further scaffolded with Hi-C data in YaHS ( Zhou *et al.*, 2023), using the --break option for handling potential misassemblies. The scaffolded assemblies were evaluated using Gfastats ( Formenti *et al.*, 2022), BUSCO ( Manni *et al.*, 2021) and MERQURY.FK ( Rhie *et al.*, 2020).

The mitochondrial genome was assembled using MitoHiFi ( Uliano-Silva *et al.*, 2023), which runs MitoFinder ( Allio *et al.*, 2020) and uses these annotations to select the final mitochondrial contig and to ensure the general quality of the sequence.

### Assembly curation

The assembly was decontaminated using the Assembly Screen for Cobionts and Contaminants ( ASCC) pipeline. TreeVal was used to generate the flat files and maps for use in curation. Manual curation was conducted primarily in PretextView and HiGlass ( Kerpedjiev *et al.*, 2018). Scaffolds were visually inspected and corrected as described by Howe *et al*. (2021). Manual corrections included 23 breaks, 99 joins, and removal of 3 haplotypic duplications. The exact order and orientation of the contigs on chromosome 1 (21.8–23.5 Mbp), chromosome 3 (19.5–21.6 Mbp) and chromosome 13 (20.5–21.2 Mbp) are unknown. The curation process is documented at https://gitlab.com/wtsi-grit/rapid-curation. PretextSnapshot was used to generate a Hi-C contact map of the final assembly.

### Assembly quality assessment

The Merqury.FK tool ( Rhie *et al.*, 2020) was run in a Singularity container ( Kurtzer *et al.*, 2017) to evaluate *k*-mer completeness and assembly quality for both haplotypes using the *k*-mer databases ( *k* = 31) computed prior to genome assembly. The analysis outputs included assembly QV scores and completeness statistics.

The genome was analysed using the BlobToolKit pipeline, a Nextflow implementation of the earlier Snakemake version ( Challis *et al.*, 2020). The pipeline aligns PacBio reads using minimap2 ( Li, 2018) and SAMtools ( Danecek *et al.*, 2021) to generate coverage tracks. It runs BUSCO ( Manni *et al.*, 2021) using lineages identified by querying the GoaT database ( Challis *et al.*, 2023). For the three domain-level lineages, BUSCO genes are aligned to the UniProt Reference Proteomes database ( Bateman *et al.*, 2023) using DIAMOND blastp ( Buchfink *et al.*, 2021). The genome is divided into chunks based on the density of BUSCO genes from the closest taxonomic lineage, and each chunk is aligned to the UniProt Reference Proteomes database with DIAMOND blastx. Sequences without hits are chunked using seqtk and aligned to the NT database with blastn ( Altschul *et al.*, 1990). The BlobToolKit suite consolidates all outputs into a blobdir for visualisation. The BlobToolKit pipeline was developed using nf-core tooling ( Ewels *et al.*, 2020) and MultiQC ( Ewels *et al.*, 2016), with package management via Conda and Bioconda ( Grüning *et al.*, 2018), and containerisation through Docker ( Merkel, 2014) and Singularity ( Kurtzer *et al.*, 2017).

## Genome sequence report

### Sequence data

**Figure 2.**
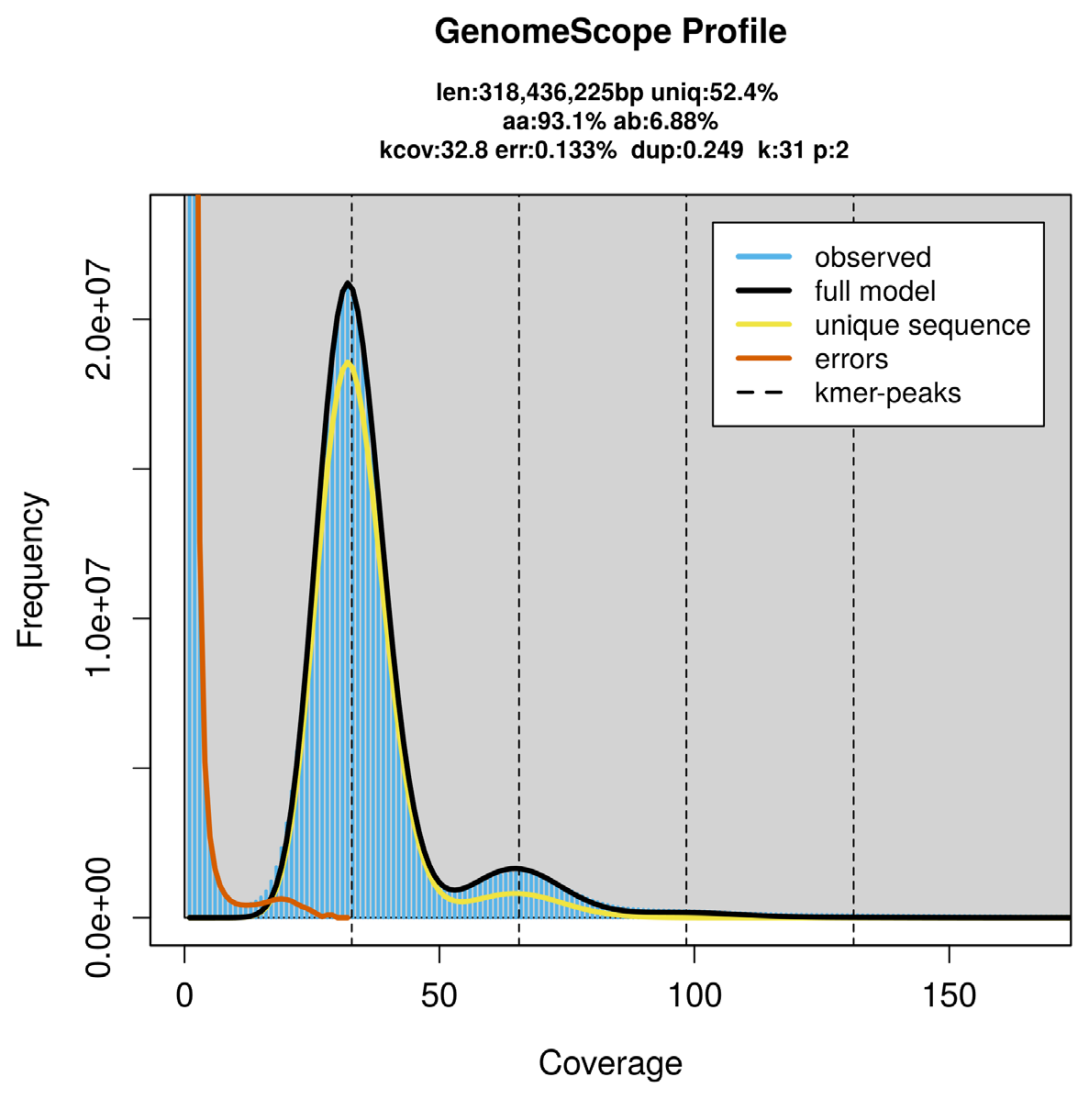
Frequency distribution of *k*-mers generated using GenomeScope2. The plot shows observed and modelled *k*-mer spectra, providing estimates of genome size, heterozygosity, and repeat content based on unassembled sequencing reads.

The genome of a specimen of *Arabis hirsuta* was sequenced using Pacific Biosciences single-molecule HiFi long reads, generating 21.83 Gb (gigabases) from 1.99 million reads, which were used to assemble the genome. GenomeScope2.0 analysis estimated the haploid genome size at 318.44 Mb, with a heterozygosity of 6.88% and repeat content of 47.71% ( Figure 2). Using flow cytometry, the genome size (1C‐value) of the sample was estimated to be 0.71 pg, equivalent to 700.00 Mb. These estimates guided expectations for the assembly. Based on the estimated genome size, the sequencing data provided approximately 66× coverage. Hi-C sequencing produced 108.36 Gb from 717.61 million reads, which were used to scaffold the assembly. Table 1 summarises the specimen and sequencing details.

**Table 1.** Specimen and sequencing data for BioProject PRJEB73956.

Platform	PacBio HiFi	Hi-C
**ToLID**	ddAraHirs1	ddAraHirs1
**Specimen ID**	EDTOL05514	EDTOL05514
**BioSample (source individual)**	SAMEA114214493	SAMEA114214493
**BioSample (tissue)**	SAMEA114214588	SAMEA114214588
**Tissue**	leaf	leaf
**Sequencing platform and model**	Sequel IIe	Illumina NovaSeq X
**Run accessions**	ERR12760840	ERR12765213
**Read count total**	1.99 million	717.61 million
**Base count total**	21.83 Gb	108.36 Gb

### Assembly statistics

The genome was assembled into two haplotypes using Hi-C phasing. Haplotype 1 was curated to chromosome level, while haplotype 2 was assembled to scaffold level. The final assembly has a total length of 582.67 Mb in 22 scaffolds, with 408 gaps, and a scaffold N50 of 37.6 Mb ( Table 2).

**Table 2.** Genome assembly statistics.

Assembly name	ddAraHirs1.hap1.1	ddAraHirs1.hap2.1
**Assembly accession**	GCA_964056655.1	GCA_964056645.1
**Assembly level**	chromosome	scaffold
**Span (Mb)**	582.67	576.85
**Number of chromosomes**	16	N/A
**Number of contigs**	430	554
**Contig N50**	2.63 Mb	2.64 Mb
**Number of scaffolds**	22	257
**Scaffold N50**	37.6 Mb	37.1 Mb
**Longest scaffold length (Mb)**	52.66	N/A
**Organelles**	Mitochondrial genome: 342.94 kb	N/A

**Figure 3.**
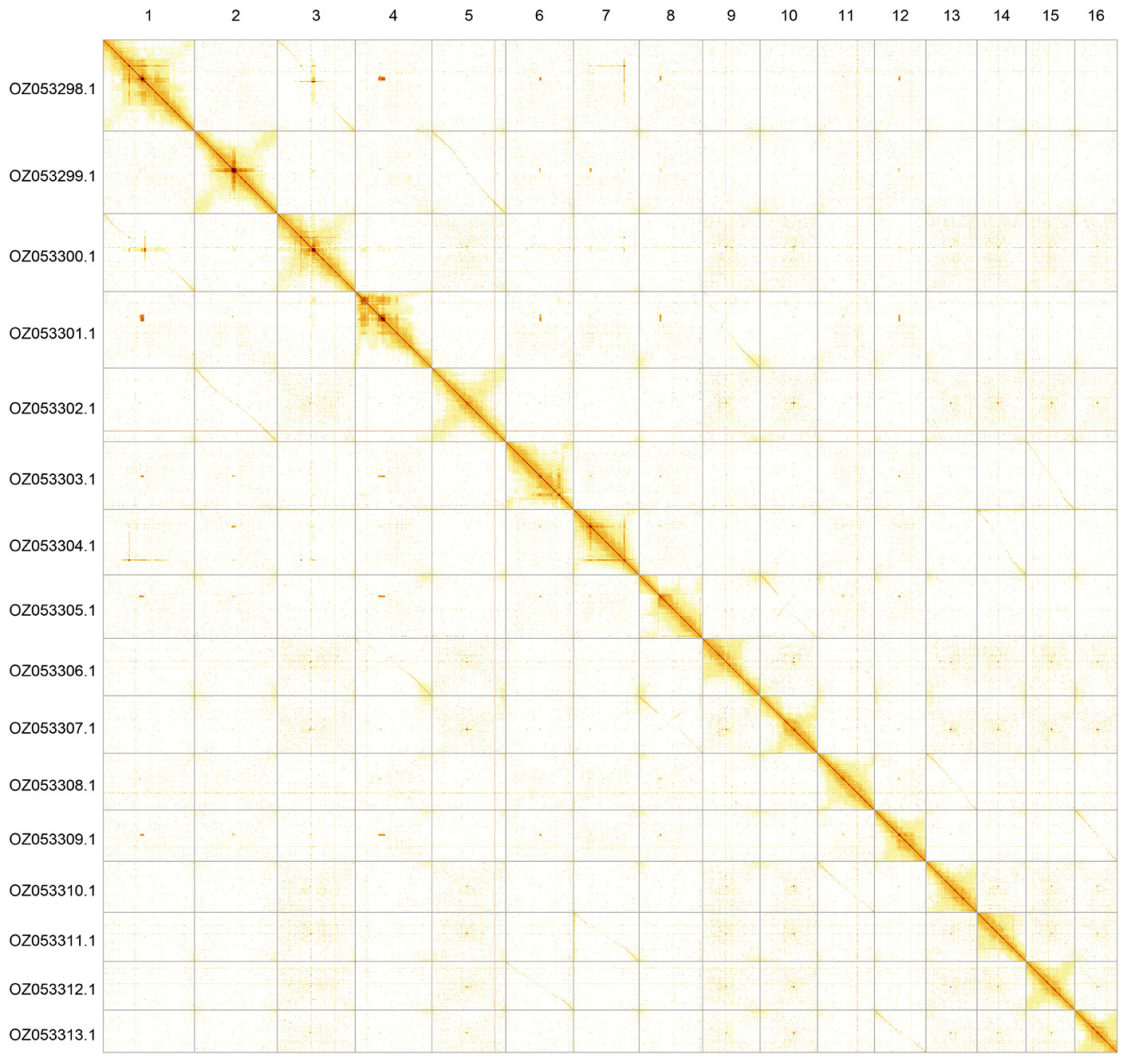
Hi-C contact map of the *Arabis hirsuta* genome assembly. Assembled chromosomes are shown in order of size and labelled along the axes. The plot was generated using PretextSnapshot.

Most of the assembly sequence (99.96%) was assigned to 16 chromosomal-level scaffolds. These chromosome-level scaffolds, confirmed by Hi-C data, are named according to size ( Figure 3; Table 3).

**Table 3.** Chromosomal pseudomolecules in the haplotype 1 genome assembly of *Arabis hirsuta* ddAraHirs1.

INSDC accession	Molecule	Length (Mb)	GC%
OZ053298.1	1	52.66	38.50
OZ053299.1	2	47.53	37
OZ053300.1	3	44.67	38
OZ053301.1	4	44.09	38
OZ053302.1	5	42.36	36.50
OZ053303.1	6	38.92	38
OZ053304.1	7	37.60	38
OZ053305.1	8	36.50	37
OZ053306.1	9	33.10	37
OZ053307.1	10	32.97	36.50
OZ053308.1	11	32.68	37.50
OZ053309.1	12	29.53	37.50
OZ053310.1	13	29.38	37
OZ053311.1	14	28.06	36.50
OZ053312.1	15	28.01	36.50
OZ053313.1	16	24.37	36.50
OZ053314.1	MT	0.34	45
OZ053315.1	Pltd	0.15	36.50

The mitochondrial and plastid genomes were also assembled. These sequences are included as contigs in the multifasta file of the genome submission and as standalone records.

### Assembly quality metrics

**Figure 4.**
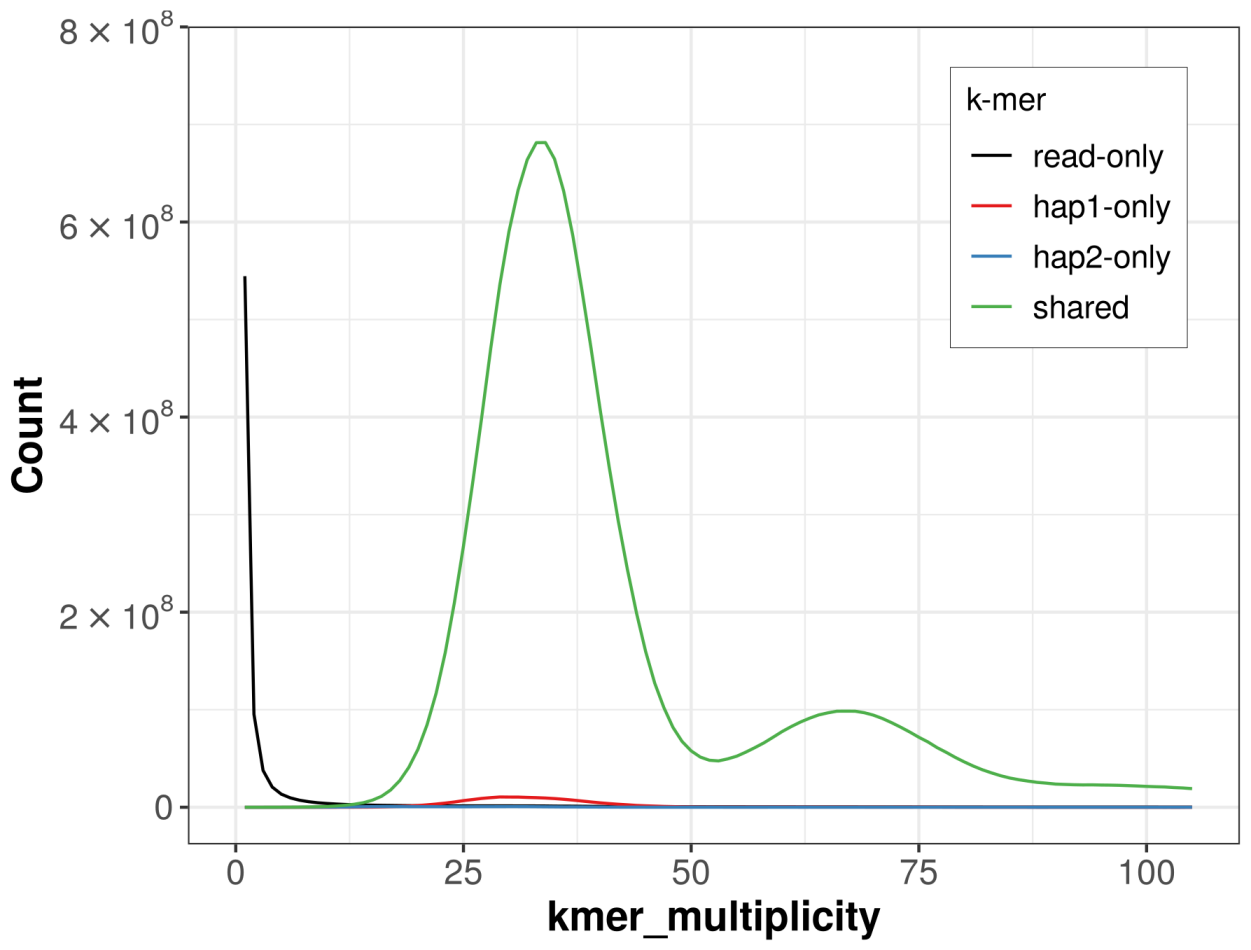
Evaluation of *k*-mer completeness using MerquryFK. This plot illustrates the recovery of *k*-mers from the original read data in the final assemblies. The horizontal axis represents *k*‐mer multiplicity, and the vertical axis shows the number of *k*-mers. The black curve represents *k*-mers that appear in the reads but are not assembled. The green curve (the homozygous peak) corresponds to *k*-mers shared by both haplotypes and the red and blue curves (the heterozygous peaks) show *k*-mers found only in one of the haplotypes.

For haplotype 1, the estimated QV is 68.1, and for haplotype 2, the QV is 63.9. When the two haplotypes are combined, the assembly achieves an estimated QV of 65.5. The *k*-mer completeness is 99.19% for haplotype 1, 97.97% for haplotype 2, and 99.41% for the combined haplotypes ( Figure 4).

**Figure 5.**
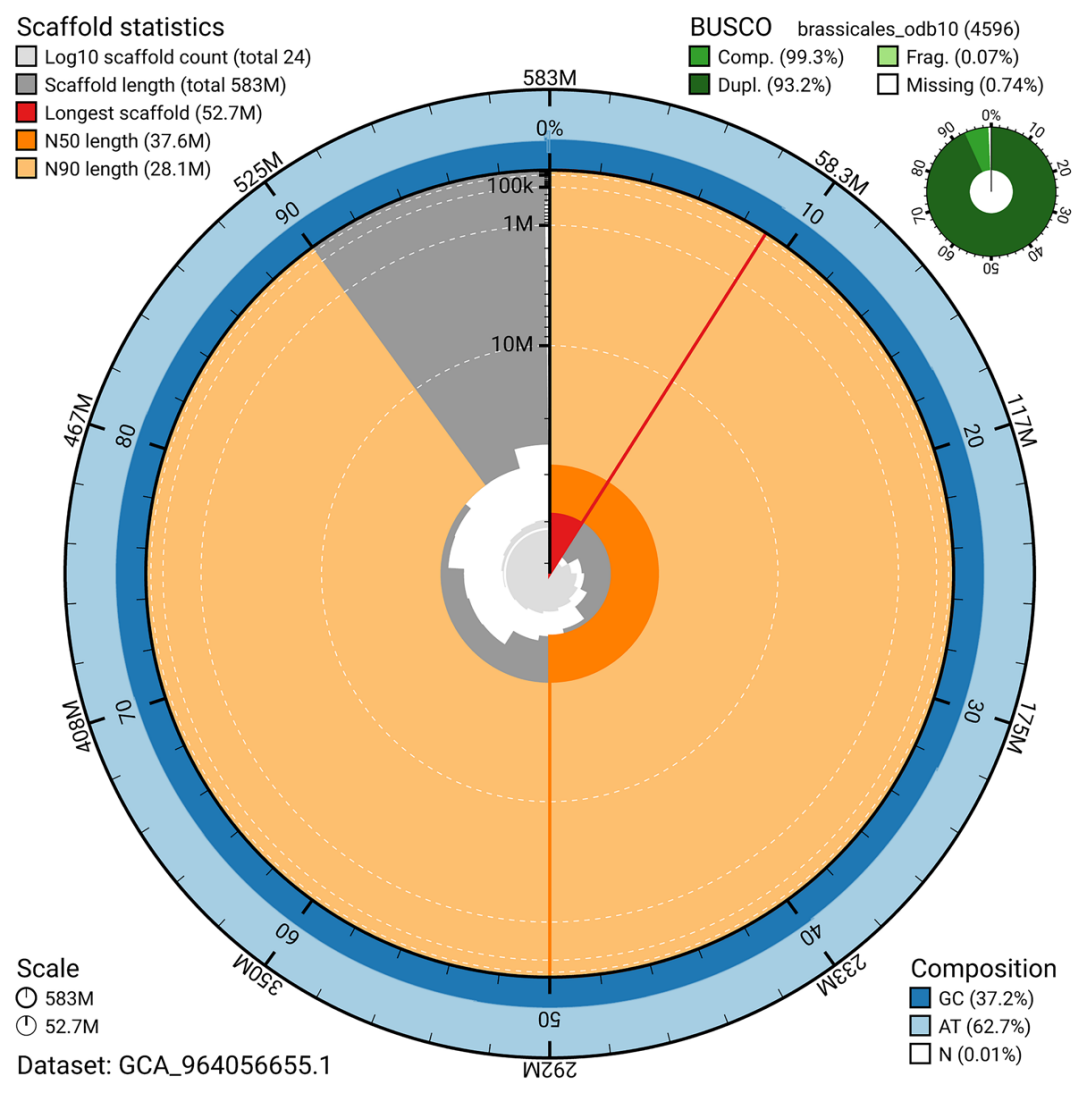
Assembly metrics for ddAraHirs1.hap1.1. The BlobToolKit snail plot provides an overview of assembly metrics and BUSCO gene completeness. The circumference represents the length of the whole genome sequence, and the main plot is divided into 1,000 bins around the circumference. The outermost blue tracks display the distribution of GC, AT, and N percentages across the bins. Scaffolds are arranged clockwise from longest to shortest and are depicted in dark grey. The longest scaffold is indicated by the red arc, and the deeper orange and pale orange arcs represent the N50 and N90 lengths. A light grey spiral at the centre shows the cumulative scaffold count on a logarithmic scale. A summary of complete, fragmented, duplicated, and missing BUSCO genes in the set is presented at the top right. An interactive version of this figure can be accessed on the BlobToolKit viewer.

**Figure 6.**
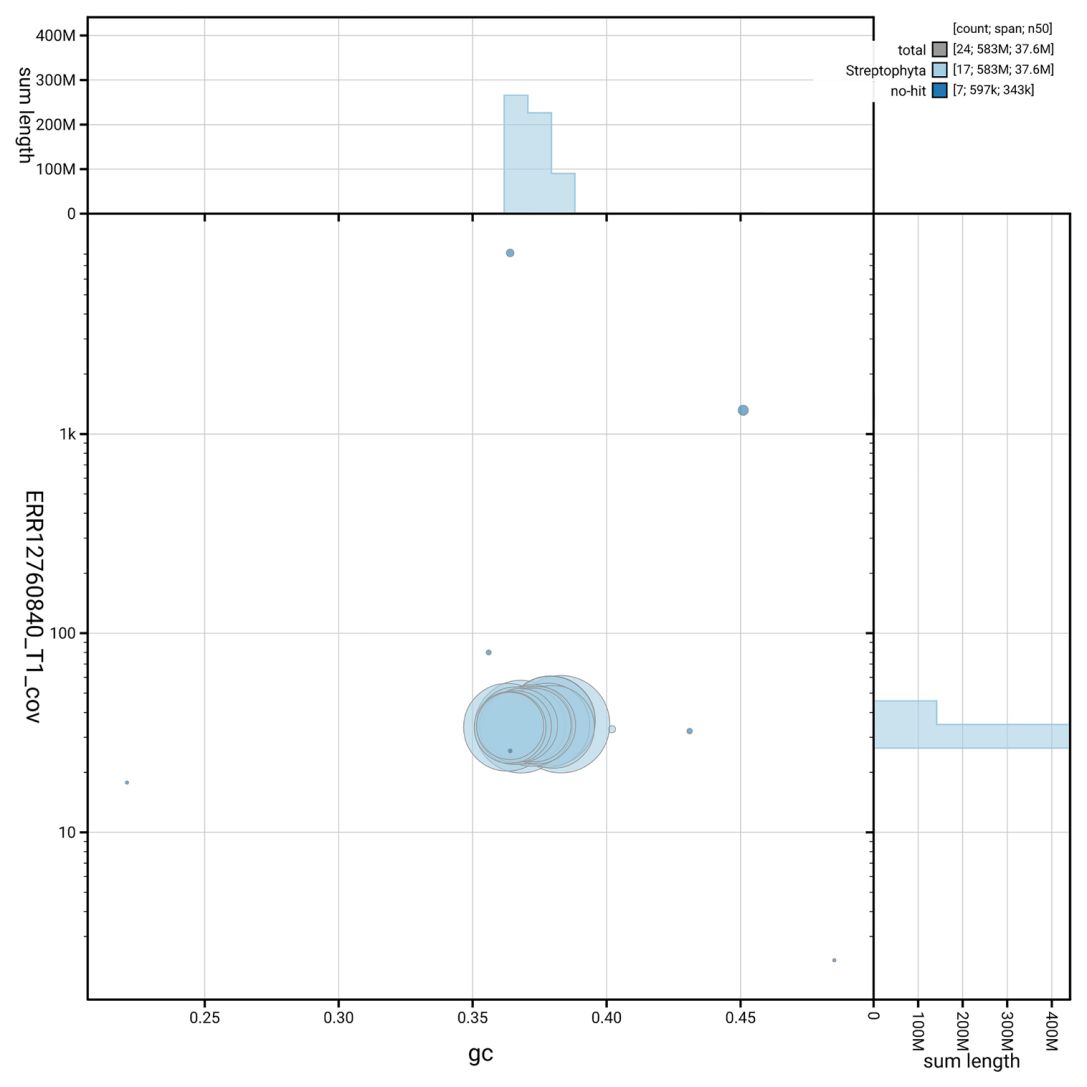
BlobToolKit GC-coverage plot for ddAraHirs1.hap1.1. Blob plot showing sequence coverage (vertical axis) and GC content (horizontal axis). The circles represent scaffolds, with the size proportional to scaffold length and the colour representing phylum membership. The histograms along the axes display the total length of sequences distributed across different levels of coverage and GC content. An interactive version of this figure is available on the BlobToolKit viewer.

BUSCO v.5.5.0 analysis using the brassicales_odb10 reference set ( *n* = 4,596) identified 99.3% of the expected gene set (single = 6.0%, duplicated = 93.2%) for haplotype 1. The snail plot in Figure 5 summarises the scaffold length distribution and other assembly statistics for haplotype 1. The blob plot in Figure 6 shows the distribution of scaffolds by GC proportion and coverage for haplotype 1.

Table 4 lists the assembly metric benchmarks adapted from Rhie *et al.* (2021) the Earth BioGenome Project Report on Assembly Standards September 2024. The EBP metric calculated for the haplotype 1 is **6.C.Q63**, meeting the recommended reference standard.

**Table 4.** Earth Biogenome Project summary metrics for the *Arabis hirsuta* assembly.

Measure	Value	Benchmark
EBP summary (haplotype 1)	6.C.Q63	6.C.Q40
Contig N50 length	2.63 Mb	≥ 1 Mb
Scaffold N50 length	37.60 Mb	= chromosome N50
Consensus quality (QV)	Haplotype 1: 68.1; haplotype 2: 63.9; combined: 65.5	≥ 40
*k*-mer completeness	Haplotype 1: 99.19%; Haplotype 2: 97.97%; combined: 99.41%	≥ 95%
BUSCO	C:99.3%[S:6.0%;D:93.2%]; F:0.1%; M:0.7%; n:4 596	S > 90%; D < 5%
Percentage of assembly assigned to chromosomes	99.96%	≥ 90%

### Wellcome Sanger Institute – Legal and Governance

The materials that have contributed to this genome note have been supplied by a Darwin Tree of Life Partner. The submission of materials by a Darwin Tree of Life Partner is subject to the **‘Darwin Tree of Life Project Sampling Code of Practice’**, which can be found in full on the [Darwin Tree of Life website] ( https://www.darwintreeoflife.org/project-resources/). By agreeing with and signing up to the Sampling Code of Practice, the Darwin Tree of Life Partner agrees they will meet the legal and ethical requirements and standards set out within this document in respect of all samples acquired for, and supplied to, the Darwin Tree of Life Project. Further, the Wellcome Sanger Institute employs a process whereby due diligence is carried out proportionate to the nature of the materials themselves, and the circumstances under which they have been/are to be collected and provided for use. The purpose of this is to address and mitigate any potential legal and/or ethical implications of receipt and use of the materials as part of the research project, and to ensure that in doing so we align with best practice wherever possible. The overarching areas of consideration are:

- Ethical review of provenance and sourcing of the material
- Legality of collection, transfer and use (national and international)

Each transfer of samples is further undertaken according to a Research Collaboration Agreement or Material Transfer Agreement entered into by the Darwin Tree of Life Partner, Genome Research Limited (operating as the Wellcome Sanger Institute), and in some circumstances, other Darwin Tree of Life collaborators.

## Data Availability

European Nucleotide Archive: Arabis hirsuta. Accession number PRJEB73956. The genome sequence is released openly for reuse. The *Arabis hirsuta* genome sequencing initiative is part of the Darwin Tree of Life Project (PRJEB40665) and Sanger Institute Tree of Life Programme (PRJEB43745). All raw sequence data and the assembly have been deposited in INSDC databases. The genome will be annotated using available RNA-Seq data and presented through the Ensembl pipeline at the European Bioinformatics Institute. Raw data and assembly accession identifiers are reported in Table 1 and Table 2. Pipelines used for genome assembly at the WSI Tree of Life are available at https://pipelines.tol.sanger.ac.uk/pipelines. Table 5 lists software versions used in this study.

## Author information

The following author collectives

- Members of the Royal Botanic Garden Edinburgh Genome Acquisition Lab
- Members of the Plant Genome Sizing collective
- Members of the Wellcome Sanger Institute Tree of Life Management, Samples and Laboratory team
- Members of Wellcome Sanger Institute Scientific Operations – Sequencing Operations
- Members of the Wellcome Sanger Institute Tree of Life Core Informatics team
- Members of the Tree of Life Core Informatics collective
- Members of the Darwin Tree of Life Consortium
