# Supplementary material for: The genome sequence of the hairy rockcress, Arabis hirsuta (L.) Scop. (Brassicales: Brassicaceae)
Source: Wellcome Open Res. 2025 Jul 25;10:365. [Version 1] doi: 10.12688/wellcomeopenres.24571.1 (PMC12616124; doi:10.12688/wellcomeopenres.24571.1)
Supplement: Peer review history

# Reviewer report: Reviewer response for version 1

**DOI:** 10.21956/wellcomeopenres.27080.r137722

**Published:** 2025-11-13

**Authors:** Laura Leventhal

**Affiliations:** 1 Integrative Biology, Stanford University, Berkeley, California, USA

The authors assembled the *Arabis hirsuta* genome using appropriate sequencing and library prep protocols, and they report a thorough set of quality metrics that demonstrate the assembly’s completeness and accuracy. The data and code are both available and accessible, and the software and relevant links are available in a table. I second the suggestion that the authors could clarify if the parameters of the software used differed from the standard parameters. 

 I enjoyed reading about some of the biological background in the introduction; however, as a minor point, I wish some of the results of the genome were compared with well known genomes in the mustard family such as *Arabidopsis thaliana*, *Arabidopsis lyrata*, or *Boechera stricta*. It would be interesting to incorporate synteny plots or discussion of chromosome structure to help the readers understand this assembly within the broader context of Brassicaceae genomic diversity. Overall, I enjoyed the manuscript and look forward to seeing these additional perspectives discussed if possible in this or future publications with the *A. hirsuta* genome.

Are sufficient details of methods and materials provided to allow replication by others?

Yes

Is the rationale for creating the dataset(s) clearly described?

Yes

Are the datasets clearly presented in a useable and accessible format?

Yes

Are the protocols appropriate and is the work technically sound?

Yes

Reviewer Expertise:

Genetics, plant genomics, Arabidopsis thaliana, evolution, evolutionary ecology

I confirm that I have read this submission and believe that I have an appropriate level of expertise to confirm that it is of an acceptable scientific standard.

# Reviewer report: Reviewer response for version 1

**DOI:** 10.21956/wellcomeopenres.27080.r128583

**Published:** 2025-08-29

**Authors:** Francesco Garassino

**Affiliations:** 1 University of Zurich, Zürich, Zurich, Switzerland

Ruhsam, Hollingsworth et al. clearly and concisely present an assembly for *Arabis irsuta*. Due to the complex phylogenetics of the Brassicaceae family, the genome sequencing of its members remains an important scientific contribution. The genome assembly presented in this Note appears to be of good quality and to have been generated through sound and well-documented methodology.

 Two minor observations on the methods' description:

 1. While the software and pipelines employed are well reported and made available to readers, it is unclear whether those were run using standard parameters and/or options. I'd encourage the authors to clarify this, if possible.

 2. Style-related: different Methods paragraph deal differently with manufacturer identification (eg: while Pacific Biosciencens is first introduced as "(Pacific Biosciences, California, USA)", Anima Genomics is simply mentioned as such). I'd suggest revising all manufacturer information to ensure consistency.

 Besides these minor observations, the Data Note meets the standards of Wellcome Open Research and is an interesting addition to its collection.

Are sufficient details of methods and materials provided to allow replication by others?

Yes

Is the rationale for creating the dataset(s) clearly described?

Yes

Are the datasets clearly presented in a useable and accessible format?

Yes

Are the protocols appropriate and is the work technically sound?

Yes

Reviewer Expertise:

Plant Genomics and Transcriptomics, Plant Ecophysiology

I confirm that I have read this submission and believe that I have an appropriate level of expertise to confirm that it is of an acceptable scientific standard.
